# An RNF168 fragment defective for focal accumulation at DNA damage is proficient for inhibition of homologous recombination in BRCA1 deficient cells

**DOI:** 10.1093/nar/gku421

**Published:** 2014-05-14

**Authors:** Meilen C. Muñoz, Diana A. Yanez, Jeremy M. Stark

**Affiliations:** 1 Department of Radiation Biology, Beckman Research Institute of the City of Hope, 1500 E Duarte Road, Duarte, CA 91010, USA; 2 Irell and Manella Graduate School of Biological Sciences, Beckman Research Institute of the City of Hope, 1500 E Duarte Road, Duarte, CA 91010, USA

## Abstract

The E3 ubiquitin ligase RNF168 is a DNA damage response (DDR) factor that promotes monoubiquitination of H2A/H2AX at K13/15, facilitates recruitment of other DDR factors (e.g. 53BP1) to DNA damage, and inhibits homologous recombination (HR) in cells deficient in the tumor suppressor BRCA1. We have examined the domains of RNF168 important for these DDR events, including chromosomal HR that is induced by several nucleases (I-SceI, CAS9-WT and CAS9-D10A), since the inducing nuclease affects the relative frequency of distinct repair outcomes. We found that an N-terminal fragment of RNF168 (1-220/N221*) efficiently inhibits HR induced by each of these nucleases in BRCA1 depleted cells, and promotes recruitment of 53BP1 to DNA damage and H2AX monoubiquitination at K13/15. Each of these DDR events requires a charged residue in RNF168 (R57). Notably, RNF168-N221* fails to self-accumulate into ionizing radiation induced foci (IRIF). Furthermore, expression of RNF168 WT and N221* can significantly bypass the role of another E3 ubiquitin ligase, RNF8, for inhibition of HR in BRCA1 depleted cells, and for promotion of 53BP1 IRIF. We suggest that the ability for RNF168 to promote H2A/H2AX monoubiquitination and 53BP1 IRIF, but not RNF168 self-accumulation into IRIF, is important for inhibition of HR in BRCA1 deficient cells.

## INTRODUCTION

RING finger nuclear factor 168 (RNF168) is an E3 ubiquitin ligase that has emerged as a key factor in the DNA damage response (DDR). For one, RNF168 genetic deficiency in human patients and mice causes radio-sensitivity and immune deficiency linked to reduced class switch recombination during antibody maturation, which involves end joining repair of programmed double strand breaks (DSBs) (1–3). RNF168 is also important for accumulation of a number of downstream DDR factors into ionizing radiation-induced foci (IRIF), including 53BP1 and BRCA1 (4–6). The influence of RNF168 on DSB repair is similar to 53BP1, in that 53BP1 is also important for class switch recombination (7), and both 53BP1 and RNF168 inhibit homologous recombination (HR) repair of DSBs, particularly in BRCA1 deficient cells (8–10). Furthermore, RNF168 promotes a ubiquitination event that facilitates 53BP1 recruitment. Namely, RNF168 promotes ubiquitination of N-terminal lysines (K13/15) on histone H2A and the variant H2AX (11,12), and ubiquitinated K15 has been shown to facilitate direct binding of 53BP1 to nucleosomes (13). Thus, characterizing how RNF168 facilitates these distinct DDR events, and how RNF168 itself is regulated during the DDR, is important for understanding the mechanisms of genome maintenance.

One means of investigating RNF168 regulation is to evaluate its focal accumulation at chromosomal breaks, such as measuring IRIF of RNF168. By this assay, RNF168 IRIF are promoted by another E3 ubiquitin ligase, RNF8, which is recruited to chromosomal breaks via MDC1 bound to a phosphorylated form of the histone variant H2AX that is induced at DSBs (γH2AX) (5,6,14–17). Furthermore, RNF168 appears to promote self-accumulation into IRIF (18), whereas other factors that promote degradation of RNF168 (i.e. TRIP12 and UBR5) limit the formation of RNF168 IRIF (19,20). While such studies have provided key insight into the regulation of RNF168 IRIF, the relative requirement of RNF168 focal accumulation for its downstream DDR functions, such as inhibition of HR, has been unclear. Along these lines, while RNF8 is critical for accumulation of RNF168 into IRIF, some functions of RNF168 do not appear to be absolutely dependent on RNF8. In reactions with purified proteins, RNF168 promotes ubiquitination of the N-terminal residues on nucleosomal H2A/H2AX, in a manner that is independent of prior ubiquitination of nucleosomes via RNF8 (11,13). Furthermore, elevated expression of RNF168 has been shown to bypass the requirement of RNF8 for 53BP1 IRIF formation (6). In contrast, 53BP1 IRIF appear dependent on a charged residue in RNF168 (R57) that is also important for ubiquitination of H2A/H2AX on K13/15 (11).

Using a functional analysis of RNF168 domains, we have sought to evaluate the relative requirement for RNF168 accumulation into IRIF, and the importance of the charge at R57, for RNF168 downstream DDR functions, with a particular focus on inhibition of HR in BRCA1 deficient cells. The HR genome maintenance pathway is critical for tumor suppression, and furthermore likely influences tumor therapeutic response to clastogenic agents (21,22). In particular, inherited mutations in the HR mediators *BRCA1* and *BRCA2* are associated with increased risk of breast and ovarian cancer, and *BRCA*-mutated ovarian cancers show distinct therapeutic responses (23). We have focused on the role of RNF168 during HR in BRCA1 deficient cells because of the relevance of BRCA1 loss to cancer etiology, and also since RNF168 has a much more substantial effect on HR in BRCA1-deficient cells (8), as has been found with 53BP1 (9,10,24). For this analysis of HR, we have developed reagents to examine HR induced by several distinct nucleases (I-SceI, CAS9-WT and CAS9-D10A). We find that RNF168 is important for inhibition of HR induced by each of these nucleases in BRCA1 deficient cells, indicating that its role in HR is not limited to one specific type of chromosomal break. Next, we show that the charge of RNF168-R57 is important for several DDR functions, including inhibition of HR, promotion of 53BP1 IRIF and ubiquitination of K13/15 on H2AX. In contrast, we find that the C-terminus of RNF168 is dispensable for these DDR functions, based on experiments expressing an N-terminal fragment (RNF168-N221*). Consistent with a role for the RNF168 C-terminus for its self-accumulation into IRIF (4–6,18,25), we find that RNF168-N221* fails to form IRIF. Furthermore, we show that expression of RNF168 and RNF168-N221* can bypass the requirement for RNF8 for inhibition of HR and promoting 53BP1 IRIF. Thus, we suggest that the R57 charge-dependent histone ubiquitination function of RNF168, but not necessarily the self-accumulation of RNF168 into IRIF, is important for inhibition of HR in BRCA1 deficient cells.

## MATERIALS AND METHODS

### Cell lines, siRNA and plasmids

**Figure 1.**
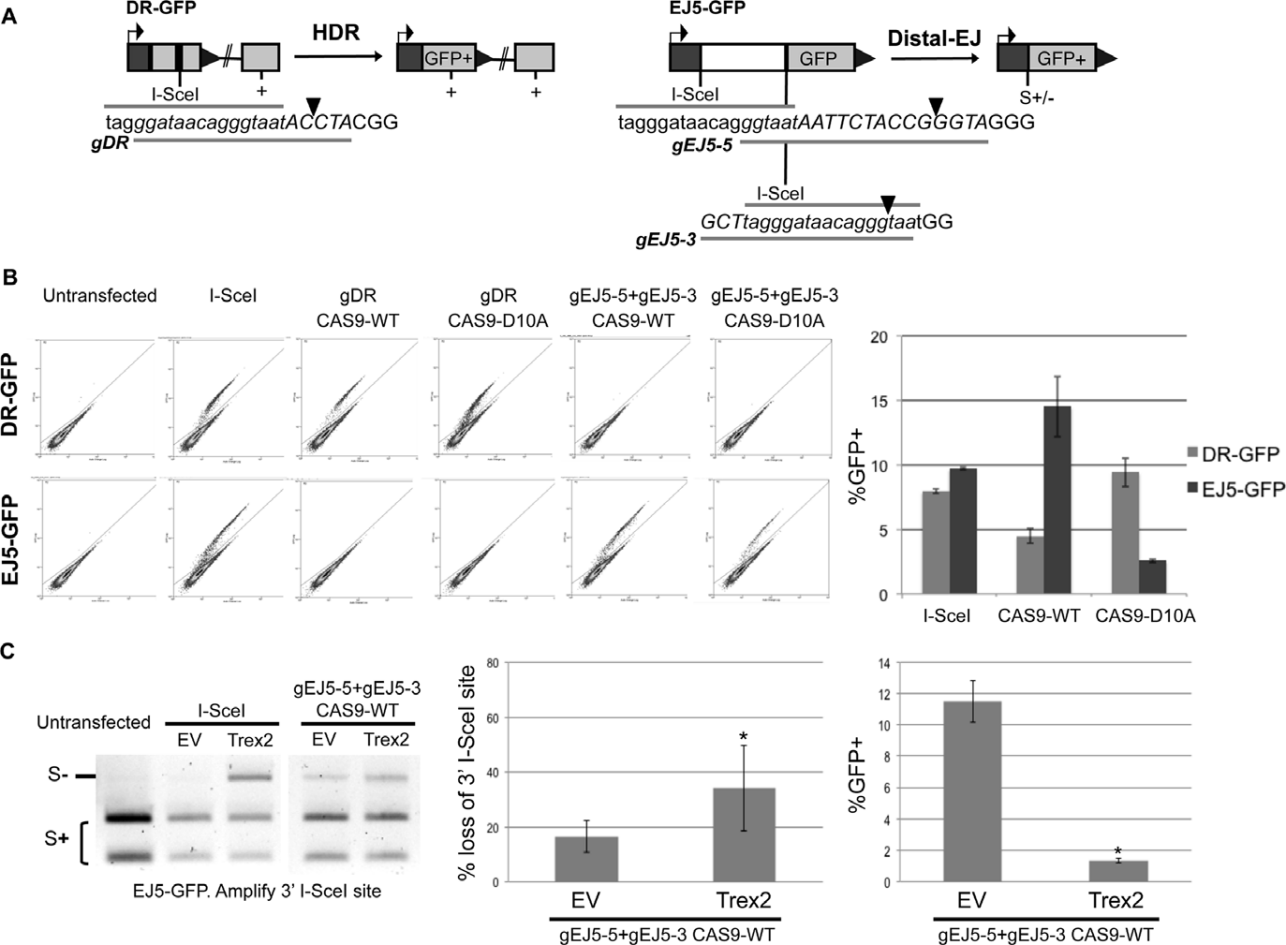
Induction of chromosomal breaks by distinct nucleases to examine HR and EJ. (**A**) Shown are diagrams of the DR-GFP and EJ5-GFP reporters for measuring HDR and Distal-EJ, respectively. Also shown are guide RNA (gRNA) targeting sequences used for inducing chromosomal breaks with the gRNA/CAS9 system (see the lines below the italicized sequence labeled gDR, gEJ5-5 and gEJ5-3), with the predicted cleavage site denoted by a triangle. Since the gRNAs include sequences flanking the I-SceI recognition site, each gRNA targets a unique site on the reporters. The I-SceI recognition site is in lower case. (**B**) Repair events induced by I-SceI, CAS9-WT and CAS9-D10A. The expression cassettes for the gRNAs and CAS9 are present on the same plasmid. U2OS cells with the DR-GFP and EJ5-GFP reporters were transfected with a set of gRNA/CAS9 plasmids, as well as I-SceI, or left untransfected. Shown are representative flow cytometry plots (FL1/green on the Y-axis, FL2 on the X-axis) from these transfections, with the GFP+ population delineated in the upper gate, used to determine the mean frequencies of GFP+ cells (*n* = 3). (**C**) CAS9-WT is proficient at inducing loss of the I-SceI site via mutagenic Proximal-EJ, which is further increased with co-expression of the exonuclease Trex2. U2OS cells with the EJ5-GFP reporter were transfected with I-SceI and CAS9-WT as in B, except including either the Trex2 expression vector or empty vector (EV). Shown are representative amplification products from these transfections digested with the I-SceI endonuclease, which were used to determine the frequency of I-SceI site loss (*n* = 6, *P* < 0.03). Also shown is the frequency of Distal-EJ from the transfections of CAS9-WT with or without Trex2 (*P < 0.0001).

U2OS cells harboring the DR-GFP, EJ5-GFP and SA-GFP reporters and the *H2ax^−/−^* mouse embryonic stem cells were previously described (26–28). The siRNAs used (Fisher/Thermoscientific, sequences as provided by the manufacturer) were siRNF168 (D-007152-18, 5′ ′GAGUAUCACUUACGCGCUA), siBRCA1 (D-003461-06 5′GGGAUACCAUGCAACAUAA or -07 5′GAAGGAGCUUUCAUCAUUC), siRNF8 (J- 006900-05 5′AGAAUGAGCUCCAAUGUAU), siFANCD2 (pool of 4 siRNAs: D-016376-01, -02, -04, -18), si53BP1 (pool of 4 siRNAs: D-003548-01, -02, -04, -05) and non-targeting siCTRL (D-001810-01). The pCAGGS-BSKX empty vector and expression vector for Flag-RNF168 resistant to siRNF168#18 was previously described (8). The R57D mutation was introduced by site-directed mutagenesis (Quikchange, Stratagene), and the N221* and R57D/N221* expression vectors were generated by amplification of the 5′ fragment of the relevant Flag-RNF168 using a 3′ primer with the N221* mutation, and insertion into pCAGGS-BSKX. Expression vectors for other mutant forms of Flag-RNF168 were generated by inserting the synthetic gene fragments (gBLOCKs, Integrated DNA Technologies) into the Flag-RNF168 full length or N221* expression vectors. To generate the siRNF8 resistant expression vector, these silent mutations (5′agaaCgaATtGcaGtgtat) were introduced into the Flag-RNF8 expression vector (29). The expression vector for H2AX was generated by isolating the coding fragment from pCDNA3-H2AX, which was generously provided by Dr. Ralph Scully (30). This fragment was inserted in frame of a 5′ Hemagglutinin tag into the pCAGGS expression vector. The K13/15Q and K118/119Q mutations in H2AX were introduced by site-directed mutagenesis (Quikchange, Stratagene). The gRNA/CAS9 plasmids were generated from pX330 (CAS9-WT version) and pX335 (CAS9-D10A version), which were generously deposited to Addgene (42230 and 42335, respectively) by Dr. Feng Zhang (31). To generate the guide RNA expression cassettes, targeting sequences shown in Figure 1 were introduced into the Bbs1 site in these plasmids.

### Transfections, IRIF analysis and DSB reporter assays

Cells were transfected similarly for the IRIF analysis and DSB reporter assays. For RNAi, 0.5 × 10^5^ U2OS cells in 0.5 ml of antibiotic-free media were plated over of a mixture of 5 pmol of each siRNA incubated with 1.8 μl of RNAiMAX (Invitrogen). Subsequently (22 h), cells were transfected with 0.1 μg of EV, RNF168 or RNF8 expression vectors using 1.8 μl Lipofectamine 2000 in 0.6 ml, and the transfection mixes were removed after 3 h.

For IRIF analysis, cells were passaged to chamber slides the day after transfection, and the following day (2 days after transfection) were treated with 6 Gy of IR (Gammacell 3000), allowed to recover for 4 h, fixed with 4% parformadehyde, quenched with 0.1-M glycine and permeabilized with 0.5% triton-X 100 prior to probing with antibodies against Flag (Sigma F3165), RNF8 (Santa Cruz Biotechnology sc-271462) and/or 53BP1 (Abcam ab36823). Two secondary antibodies (Invitrogen/Life Technologies A-11036 and A-11029) labeled with distinct fluorophores were used to distinguish signals in co-staining experiments. Images were acquired using a BX-50 (Olympus) microscope with Image-Pro software. Mean fluorescence intensity measurements were performed using Image-Pro Premier Software. Statistics were performed with the Fisher's exact test.

For the DSB reporter assays, following RNAi treatment, the I-SceI expression vector (pCBASce, 0.25 μg) or the gRNA/CAS9 plasmids (0.4 μg) were included in the Lipofectamine 2000 transfection mixes described above. For the initial characterization of the gRNA/CAS9 plasmids and comparison with I-SceI (i.e. Figure 1), cells were not treated with siRNA prior to transfection, and 0.4 μg total plasmid was transfected with Lipofectamine 2000 as above. The percentage of GFP+ cells was determined by FACS analysis (CYAN ADP, Dako) 3 days after transfection. For comparison, the repair value for each transfection is often divided by the mean repair value for the parallel control transfection (i.e., siCTRL and/or EV). Each repair value is the mean of at least three independent transfections, error bars reflect the standard deviation, and statistics were performed with the unpaired *t*-test. Error bars denote the standard deviation from the mean.

### Immunoblotting analysis

For immunoblotting analysis, cells were lysed with NETN (20 mM Tris, pH 8, 100 mM NaCl, 1 mM EDTA, 0.5% IGEPAL, 1.25 mM DTT and Roche Protease Inhibitor Mixture) using several freeze/thaw cycles, or sonication (QSonica Q800RS ultrasonic horn). Blots of these extracts were probed with antibodies against the Flag epitope (Sigma A8592), BRCA1 (Abcam, ab16780), RNF168 (Millipore, 06-1130), RNF8 (Santa Cruz Biotech sc-271462), actin (Sigma, A2066), FANCD2 (Abcam, ab2187) and HRP-conjugated secondary antibodies (Santa Cruz Biotechnology, sc-2004 and sc-2005). To examine H2AX, *H2ax^−/−^* mouse embryonic stem cells were transfected with expression vectors for RNF168 and/or H2AX using 400 ng each plasmid transfected with 7.2 μl of Lipofectamine 2000 in a 0.4 ml total volume for 3 h. Subsequent to transfection (40 h), cells were treated with 10 Gy IR (Gammacell 3000 irradiator) and allowed to recover 2.5 h prior to lysis with NETN supplemented with 10 mM *N*-Ethylmaleimide, treated with two freeze/thaw cycles, and centrifuged at 4°C for 10 min. Histones were extracted from the pellet using 0.2 N HCl for 40 min at 4°C for immunoblots that were probed with an anti-H2AX antibody (Millipore 07-627, 1:1000), and developed with an HRP-conjugated secondary antibody (Santa Cruz Biotechnology sc-2004, 1:1000). HRP signals were visualized using ECL reagent (Amersham Biosciences).

## RESULTS

### Examining repair of chromosomal breaks induced by distinct site-specific nucleases

To investigate the role of RNF168 function during HR, we first sought to develop systems to evaluate HR induced by a distinct set of site-specific nucleases: I-SceI, CAS9-WT and CAS9-D10A. I-SceI is a yeast homing endonuclease that induces a double-strand break (DSB) within an 18 bp sequence, causing 4 nt. overhangs (32). The CAS9 nuclease system is derived from an *Streptococcus pyogenes* immune response pathway that uses a guide RNA (gRNA) to target strand breaks in homologous DNA (33,34). Expression of this nuclease has been adapted to mammalian cells in a single plasmid containing two expression cassettes: one for the protein CAS9 and one for a synthetic gRNA with the target sequence at the 5′ end (33). CAS9 contains two nuclease domains that induce a blunt ended DSB that is positioned 3 nt. upstream of the 3′ end of the target sequence (33,34). The CAS9-D10A mutation disrupts the N-terminal nuclease domain, such that CAS9-D10A is proficient at nicking the DNA strand complementary to the gRNA, but is deficient at cleaving the second strand (33,34). Accordingly, CAS9-D10A induces single strand nicks rather than DSBs (33,34), although certainly such nicks could be processed into DSBs within the cell. One prediction of the reduced second-strand cleavage of CAS9-D10A is that it should be less proficient at inducing end-joining (EJ) versus HR, as compared to CAS9-WT or I-SceI.

To test this prediction, we evaluated the relative efficiency of CAS9-WT and CAS9-D10A to induce HR versus EJ, using previously described reporter systems integrated into U2OS human cells (28). For HR, we used the DR-GFP reporter, which is designed to measure a RAD51-dependent subtype of HR, homology-directed repair (HDR). DR-GFP contains a GFP expression cassette that is interrupted by an I-SceI recognition site, followed 3′ by an internal GFP fragment, which if used as a template for HDR, restores the GFP+ cassette (35). For EJ, we used the EJ5-GFP reporter, which contains two segments of a GFP expression cassette interrupted by a *puro* gene that is flanked by two I-SceI recognition sites (36). EJ that uses the distal ends of two tandem I-SceI-induced DSBs (Distal-EJ) restores the GFP expression cassette (36). We designed a set of gRNAs that target a portion of the I-SceI recognition site in each reporter along with unique flanking sequence. Accordingly, gDR is designed to target the I-SceI site in DR-GFP, while gEJ5-5 and gEJ5-3 target the 5′ and 3′ I-SceI sites in EJ5-GFP, respectively (Figure 1A). Consistent with the sequence specificity of the gRNAs to direct CAS9 chromosomal breaks, expression of CAS9-WT with gDR, but not gEJ5-5/gEJ5-3, induced the GFP+ HDR product in the DR-GFP reporter; and expression of CAS9-WT with gEJ5-5/gEJ5-3, but not gDR, induced the GFP+ Distal-EJ product in EJ5-GFP (Figure 1B). Using these reagents, we compared the relative efficiency of each nuclease at inducing HDR versus Distal-EJ. We found that I-SceI expression induced HDR slightly less than Distal-EJ (20% lower), CAS9-WT expression induced HDR significantly less than Distal-EJ (3-fold), whereas CAS9-D10A induced HDR significantly greater than Distal-EJ (3.7-fold). This latter finding is consistent with the notion that CAS9-D10A is deficient at second-strand cleavage, since HDR is predicted to be induced by both DSBs and single strand nicks (33,34), whereas Distal-EJ is likely more dependent on DSBs. Although, the readily detectable level of Distal-EJ in this experiment indicates that CAS9-D10A-induced nicks may be processed into DSBs, albeit at a relatively low frequency, which has been proposed in previous reports (33,37,38).

Given that CAS9-WT showed a bias for inducing Distal-EJ versus HDR, compared to I-SceI, we hypothesized that the DSBs induced by CAS9-WT may be more prone to mutagenic EJ, thereby causing less HDR. To test this, we quantified the frequency of loss of the I-SceI site resulting from EJ that retains proximal DSB ends (Proximal-EJ) of the 3′ I-SceI site in EJ5-GFP, using PCR and I-SceI digestion analysis. As described previously, expression of I-SceI alone causes an undetectable level of I-SceI-resistant Proximal-EJ products, whereas co-expression of I-SceI and the 3′ exonuclease Trex2 causes a substantial level of such products (39) (Figure 1C). In contrast, CAS9-WT expression alone causes significant loss of the I-SceI site during Proximal-EJ (17 ± 6%, Figure 1C), which is enhanced by co-expression of the Trex2 exonuclease (2-fold, *P* < 0.03, Figure 1C). Furthermore, co-expression of Trex2 with CAS9-WT causes a reduction in Distal-EJ (>8-fold relative to CAS9-WT alone, *P* < 0.0001), which is likely due to increased frequency of mutagenic Proximal-EJ events, as has been found with I-SceI and Trex2 (39). Sequencing analysis of individual I-SceI-resistant products from the CAS9-WT samples showed deletion mutations flanking the predicted cleavage site, and Trex2 expression did not cause an obvious increase in deletion size (Table 1). Thus, DSBs induced by CAS9-WT appear more prone to repair via mutagenic EJ, compared to I-SceI, but such mutagenic EJ is nevertheless enhanced with co-expression of Trex2. In summary, the inducing nuclease (I-SceI, CAS9-WT and CAS9-D10A) affects the relative frequency of distinct repair outcomes. We have presented these findings to support the rationale for examining this distinct set of nucleases in the HR experiments below.

**Table 1.** Sequences of I-SceI-resistant Proximal-EJ products amplified from the 3′ I-SceI site in the EJ5-GFP reporter, following the CAS9-WT and Trex2 transfections described in Figure 1C

I-SceI site in capital letters. / = predicted CAS9 cleavage site. (deleted nucleotides). bold = mutation	Incidence
**Parental sequence**	
agctTAGGGATAACAGGG/TAATggatc	
**CAS9-WT junctions**	
agctTAGGGATAAC(A)GGG/TAATggatcc	1/14
agctTAGGGATAACAG(GG)/TAATggatcc	1/14
agctTAGGGATAACA(GGG)/TAATggatc	4/14
agctTAGGGATAACAGGG/(TAATgg)atcc	1/14
agctTAGGGATAACAGGG/(TAATGG)atcc	1/14
agctTA(GGGATAACAGGG/TA)Atggatcc	2/14
agctTAGGGA**C**AA(CAGGG/TAATggatcc)ac	1/14
ag(c)tTA(GGGATAACAGGG/TAATg)gatcc	1/14
agct**A(**AGGGATAACAGGG/TAATg)gatcc	1/14
cca(tcagaagctTAGGG/ATAACAGGGTAATggatcca)ccggt	1/14
**CAS9-WT + Trex2 junctions**	
agctTAGGGATAACAG(GG/)TAATggatcc	1/11
agctTAGGGATAACAGGG/(TAATgg)atcc	1/11
agctTAGGGATAAC(AGGG/TAA)Tggatcc	1/11
agctTAGGGATAAC(AGGG/TAAT)ggatcc	1/11
agctTAGGGA(TAACAGGG/)TAATggatcc	1/11
agctTAGGG(ATAACAGGG/T)AATggatcc	1/11
agctT(AGGGATAACAGGG/)TAATggatcc	1/11
agctTA(GGGATAACAGGG/TA)Atggatcc	1/11
agctTAGG(GATAACAGGG/TAATgg)atcc	1/11
ttttg(54nt)tcgcc**g**ccat	1/11
ttcta(56nt)gtgagc	1/11

### The RNF168 N-terminus is sufficient to inhibit HR induced by multiple distinct nucleases in a manner dependent on the charge of R57

**Figure 2.**
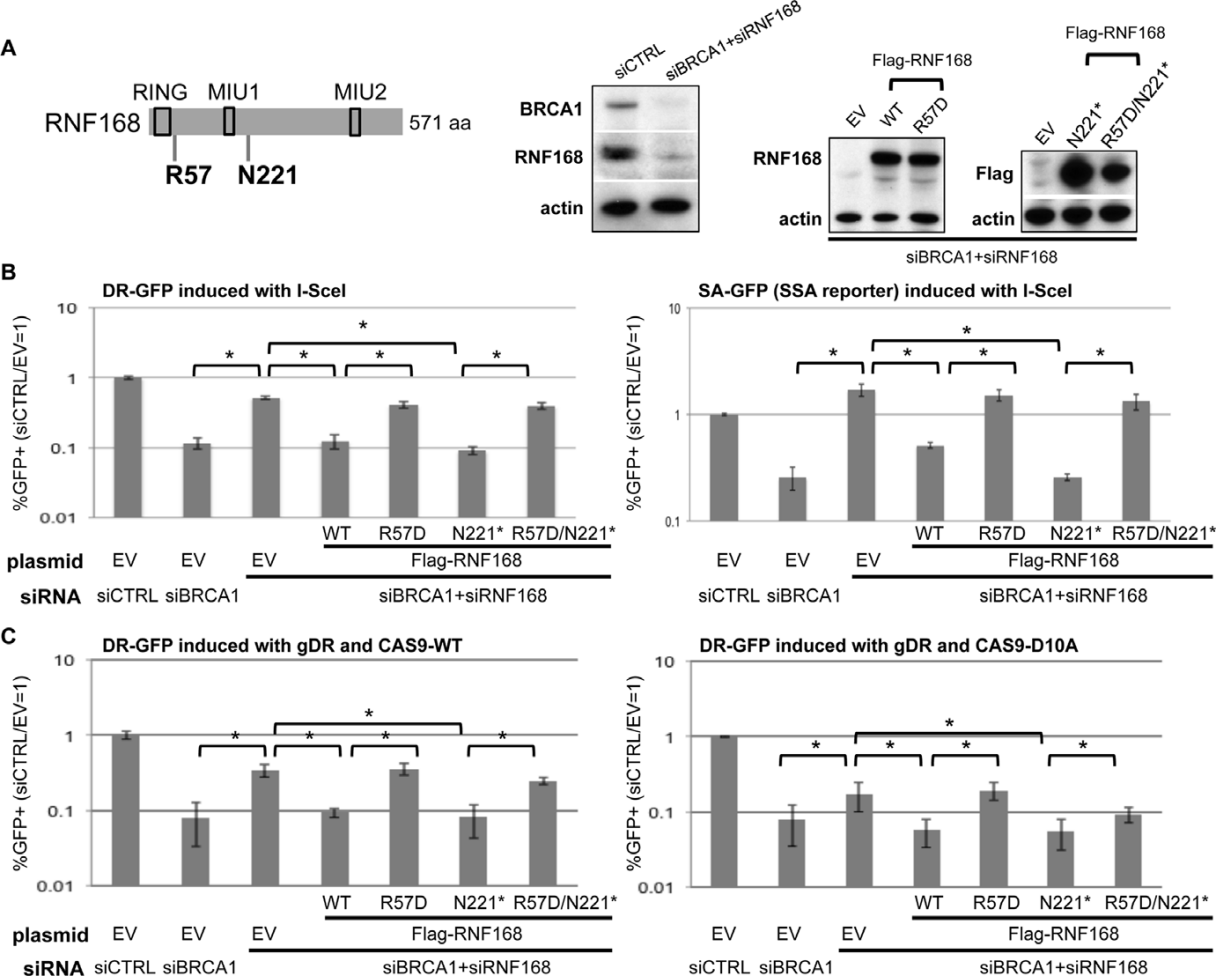
An N-terminal fragment of RNF168 (N221*) is proficient at inhibiting HR in BRCA1 deficient cells, in a manner dependent on the charge of R57. (**A**) Depletion of RNF168 and BRCA1 via siRNA and expression of RNF168 mutant forms. Shown is a diagram of RNF168 with approximate positions of the R57D and N221* mutations, as well as the RING, MIU1 and MIU2 motifs. Immunoblot signals are shown for BRCA1, RNF168 and actin for U2OS cells treated with siRNAs targeting BRCA1 and RNF168 (siBRCA1#6, siRNF168#18), and a non-targeting siRNA (siCTRL). Subsequent to siRNA treatment, cells were transfected with expression vectors for Flag-tagged RNF168 (WT, R57D, N221*, R57D/N221*, each resistant to siRNF168#18) or EV. Shown are immunoblot signals from these transfections for RNF168 (WT and R57D), Flag (N221* and R57D/N221*) and actin. (**B**) Analysis of RNF168 mutant forms for inhibition of I-SceI-induced HR in BRCA1 depleted cells. Two U2OS reporter cell lines that measure distinct types of HR were evaluated: DR-GFP that measures HDR and SA-GFP that measures SSA. These U2OS cell lines were treated with the siRNAs described in A and subsequently co-transfected with the expression vector for I-SceI along with the RNF168 expression vectors described in A, or the control EV. Shown are the frequencies of *GFP+* cells for each reporter cell line, relative to parallel transfections with a non-targeting siRNA (siCTRL) and control EV. *P < 0.0001 (*n* = 6). (**C**) RNF168 influences HDR of CAS9-induced chromosomal breaks similarly to those induced by I-SceI. U2OS DR-GFP reporter cell line was transfected as in B, except replacing I-SceI with gRNA/CAS9 plasmids expressing gDR and CAS9-WT or CAS9-D10A, as shown in Figure 1. Frequencies of *GFP+* cells were measured as in B. *P ≤ 0.022 (*n* = 6).

Using the reporter systems described above, we sought to examine the domains of RNF168 important for inhibition of HR in BRCA1 deficient cells. For these experiments, we have depleted RNF168 and BRCA1 via siRNA using U2OS reporter cell lines, followed by co-expression of each site-specific nuclease described above, along with expression of various forms of RNF168 (Figure 2A). We have evaluated two distinct HR reporters, DR-GFP (described above, Figure 1A), and SA-GFP, in which HR repair restores a GFP expression cassette via single-strand annealing (SSA). While HDR involves RAD51-dependent gene conversion, SSA is a RAD51-independent HR event resulting in a deletion between two repeats (40). Both repair events likely require end resection and are promoted by BRCA1 (40).

Using these HR assays, we evaluated a set of mutant forms of RNF168, starting with DSB induction via expression of I-SceI. As described previously, we find that BRCA1 depletion causes a significant reduction in both HDR and SSA, which can be substantially suppressed by co-depletion of RNF168 (Figure 2A and B) (8). Furthermore, this suppression is reversed by transient expression of RNF168-WT that is resistant to siRNF168 (Figure 2A and B) (8). We have extended this analysis with a set of RNF168 mutants (Figure 2A, all containing a Flag immunotag). We expressed an N-terminal fragment of RNF168 (residues 1-220, RNF168-N221*), which retains the RING domain and ubiquitin binding domains, including a conserved motif interacting with ubiquitin (MIU1) (18,25). However, RNF168-N221* lacks 60% of the protein (571 aa total), including the second conserved motif interacting with ubiquitin (MIU2) (4–6). We also expressed RNF168 with a mutation in a conserved arginine to the opposite charge (R57D), which we evaluated in full length RNF168 and in N221*. The R57D mutation does not disrupt the RING domain *per se*, in that RNF168-R57D has been shown to retain ubiquitin ligase activity; however the R57D protein is deficient at ubiquitinating histones H2A/H2AX in the context of nucleosomes (11). Expression of each of these mutant forms of RNF168 was confirmed by immunoblotting analysis (Figure 2A). From these experiments, we found that even though RNF168-N221* lacks the majority of the RNF168 protein, it is at least as proficient as RNF168-WT at inhibiting HDR and SSA in BRCA1-deficient cells (Figure 2B). In contrast, neither RNF168-R57D nor RNF168-N221*/R57D were proficient at inhibiting HR (Figure 2B).

We next performed the HDR experiments using the CAS9/gDR expression system (CAS9-WT and CAS9-D10A) instead of I-SceI, and found similar results with each nuclease. Namely, for both CAS9-WT and CAS9-D10A, BRCA1 depletion caused a decrease in HDR that was suppressed by co-depletion of RNF168, which was reversed by transient expression of RNF168-WT and RNF168-N221*, but not RNF168-R57D nor RNF168-N221*/R57D (Figure 2C). Combined, these findings indicate that an N-terminal fragment of RNF168 is proficient at inhibiting HR in a manner that is dependent on the charge of residue R57. Furthermore, the influence of RNF168 and BRCA1 on HDR appears to be conserved for repair of multiple distinct types of chromosomal breaks.

### An N-terminal fragment of RNF168 is deficient at forming IRIF, but is proficient at promoting 53BP1-IRIF and monoubiquitination of H2AX at K13/15

**Figure 3.**
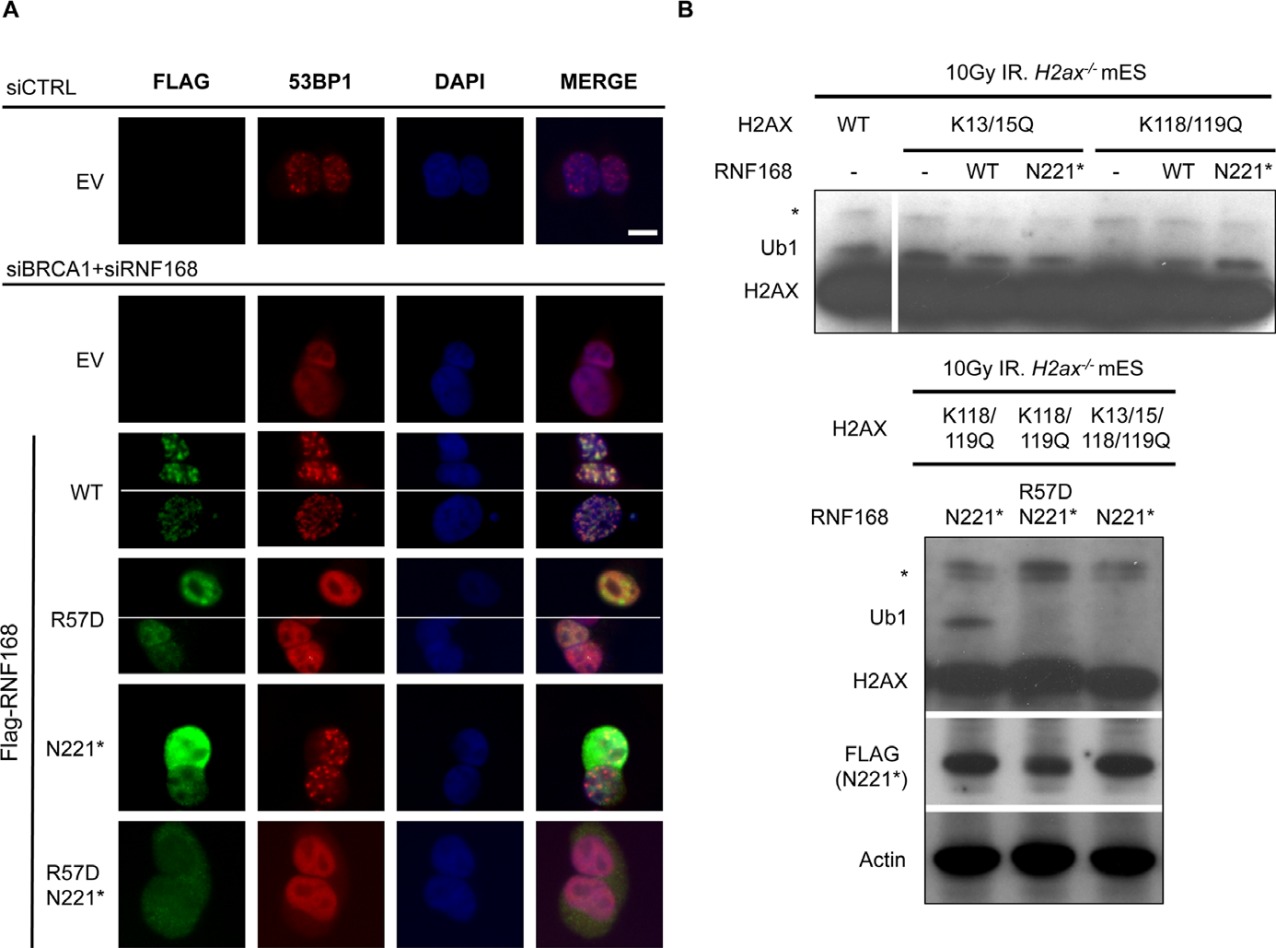
RNF168-N221* is deficient at forming IRIF, but proficient at promoting 53BP1 IRIF and ubiquitination of H2AX at K13/15, in a manner dependent on the charge of R57. (**A**) Analysis of RNF168 mutants for proficiency at forming IRIF and promoting 53BP1 IRIF. U2OS cells were treated with siCTRL or siBRCA1 + siRNF168 and subsequently transfected with each of the Flag-tagged RNF168 expression vectors described in Figure 2A. Subsequently, cells were treated with 6 Gy of IR (Cs137), and allowed to recover 4 h prior to fixation and co-immunostaining with Flag and 53BP1 antibodies. Shown are Flag and 53BP1 immunostaining, and DAPI staining for representative cells from each transfection. Scale bar = 10 μm. (**B**) Expression of RNF168-N221* promotes H2AX ubiquitination at K13/15 in a manner dependent on the charge of R57. Mouse *H2ax^−/−^* embryonic stem cells were transfected with expression vectors for H2AX with various lysine to glutamine mutations at previously described ubiquitination sites: two double mutants (K13/15Q, K118/119Q) and one quadruple mutant (K13/15/118/119Q). We analyzed each of these mutants because K118/119 monoubiquitination is predominant; such that K13/15 monoubiquitination is only detectable in the K118/119Q mutant. Cells were also co-transfected with expression vectors for RNF168, subsequently treated with 10 Gy IR (Cs137), and allowed to recover for 2.5 h prior to both soluble protein and histone extraction in the presence of a de-ubiquitination inhibitor. Shown are immunoblotting signals for H2AX from the histone extract of these samples: *denotes a non-specific band observed in untransfected cells and Ub1 denotes migration of H2AX at a position consistent with monoubiquitination. Also shown are Flag and actin immunoblotting signals for cells expressing Flag-tagged RNF168-N221 and R57D/N221*.

The above finding that RNF168-N221* is proficient at inhibiting HR is relatively unexpected, given that this peptide lacks the majority of the protein, including the MIU2 domain that promotes RNF168 focal accumulation at DNA damage (4–6,18,25). Accordingly, we hypothesized that RNF168-N221* may be deficient at focal accumulation at DNA damage. In contrast, we predicted that RNF168-N221* may be proficient at promoting focal accumulation of 53BP1 at DNA damage, which is likely an important downstream step of RNF168-mediated inhibition of HR (8,13). For these experiments, to enable direct comparison with the HR reporter experiments, we co-depleted cells of BRCA1 and RNF168 and subsequently expressed several forms of RNF168 that contain a Flag-immunotag. Following IR treatment, we performed co-immunostaining against Flag and 53BP1, and scored Flag-positive cells for IRIF of the Flag staining itself (i.e. RNF168) and for IRIF of 53BP1. We also performed control treatments to confirm that cells treated with a non-targeting siRNA (siCTRL) showed a high frequency of 53BP1 IRIF, which were substantially lost in cells co-treated with siRNF168 and siBRCA1 (Figure 3A). From these experiments (Figure 3A, Table 2), we found that full-length RNF168 accumulates into IRIF and promotes 53BP1 IRIF. In contrast, RNF168-N221* was deficient at IRIF accumulation, but was nevertheless proficient at promoting 53BP1 IRIF. With RNF168-R57D, we found that this form showed a low frequency of accumulation into IRIF, and was deficient at promoting 53BP1 IRIF, which is consistent with previous findings (11). Finally, we found that RNF168-N221*/R57D was deficient at both focal accumulation and promoting 53BP1 IRIF. Thus, RNF168-N221*, while deficient at self-accumulation into IRIF, appears proficient at inhibiting HR and promoting 53BP1 IRIF, in a manner dependent on the charge of R57. Notably, we also analyzed cells depleted of RNF168 alone (i.e. without BRCA1 depletion) and found similar results (Supplemental Figure S1). These findings indicate that the focal accumulation of RNF168 is not essential for inhibition of HR, whereas the capacity for RNF168 to promote 53BP1 IRIF correlates with its anti-HR function.

**Table 2.** Quantification of IRIF in experiments described in Figures 3, 5 and 6

siRNA treatment and RNF168 expression	Frequency of FLAG IRIF of FLAG+ cells		Frequency of 53BP1 IRIF of FLAG+ cells		
	#>5 IRIF/ total (%)	Distinct from WT	#>5 IRIF / total (%)	Distinct from WT	Distinct from N221*
**siRNF168 + siBRCA1**					
Flag-RNF168-WT	124/158 (79)		119/158 (75)		
Flag-RNF168-R57D	25/156 (16)	*P* < 0.0001	1/156 (0.6)	*P* < 0.0001	*P* < 0.0001
Flag-RNF168-N221*	0/188 (0)	*P* < 0.0001	140/188 (75)		
Flag-RNF168-N221*/R57D	0/175 (0)	*P* < 0.0001	1/175 (0.6)	*P* < 0.0001	*P* < 0.0001
Flag-RNF168-N221-571	0/95 (0)	*P* < 0.0001	0/95 (0)	*P* < 0.0001	*P* < 0.0001
Flag-RNF168-N221* LRM1Δ	0/86 (0)	*P* < 0.0001	67/86 (78)		
Flag-RNF168-N221* LLAA-A179G	0/89 (0)	*P* < 0.0001	76/89 (85)		*P* = 0.044
Flag-RNF168-N221* LRM1Δ-LLAA-A179G	0/93 (0)	*P* < 0.0001	49/93 (53)	*P* = 0.0003	*P* < 0.0001
**siRNF8 + siBRCA1**					
Flag-RNF168-WT	94/117 (90)		92/117 (79)		P < 0.0001
Flag-RNF168-R57D	15/114 (13)	*P* < 0.0001	0/114 (0)	*P* < 0.0001	*P* < 0.0001
Flag-RNF168-N221*	2/113 (2)	*P* < 0.0001	59/113 (52)	*P* < 0.0001	
Flag-RNF168-N221*/R57D	0/114 (0)	*P* < 0.0001	4/114 (3.5)	*P* < 0.0001	*P* < 0.0001

For each condition, cells were accumulated from immunofluorescence staining of at least three independent transfections. Shown are the total cells counted from the three independent transfections. Cells with >5 Flag or 53BP1 foci were scored as Flag or 53BP1 IRIF+, respectively, and cells with ≤5 Flag or 53BP1 foci were scored as IRIF−. The Fisher's exact test was used for statistics.

A likely mechanism by which RNF168 promotes 53BP1 IRIF is via monoubiquitination of H2A/H2AX at K13/15 (11,12), since this ubiquitination event has been shown to enhance binding of 53BP1 to nucleosomes (13). Furthermore using purified proteins, this ubiquitination event has been shown to be catalyzed by an N-terminal fragment of RNF168, and to be dependent on the charge of residue R57 (11). Accordingly, we hypothesized that expression of RNF168-N221* may promote this ubiquitination event in cells. To test this, we expressed mutant forms of H2AX with several lysine to glutamine mutations at previously described ubiquitination sites (K13/15Q, K118/119Q, K13/15/118/119Q) (11,12), in *H2ax^−/−^* mouse embryonic stem cells (27), with or without expression of RNF168. These lysine residues are conserved between H2AX and H2A (11,12), but we have focused on H2AX due to the feasibility of examining mutants of this non-essential H2A variant using *H2ax^−/−^* cells. Following transfection, we treated the cells with IR, purified histones in the presence of a deubiquitinase inhibitor, and probed for distinctly migrating isoforms of H2AX by immunoblotting analysis. We found that H2AX shows a prominent monoubiquitinated form that is dependent on K118/K119 (i.e. present in WT and K13/15Q, but absent in K118/119Q, Figure 3B, Lanes 1–5), which is consistent with previous reports (11,12). Thus, to evaluate the influence of RNF168 on the less frequent K13/15 monoubiquitination event, we examined the K118/119Q form of H2AX. Consistent with the notion that K13/15 monoubiquitination is infrequent, we found that monoubiquitination of K118/119Q H2AX was below the limit of detection (Figure 3B, Lane 5). However, we found that expression of either RNF168-WT or RNF168-N221* resulted in detectable levels of monoubiquitinated K118/119Q H2AX (Figure 3B, Lanes 5–7). We also found that while RNF168-N221* expression led to significant levels of monoubiquitinated H2AX K118/119Q, RNF168-N221*/R57D did not, and RNF168-N221* expression had no effect on the gel mobility of H2AX K13/15/118/119Q (Figure 3B, lower panel). These findings indicate that RNF168-N221* is proficient at promoting monoubiquitination of H2AX on K13/15 in a manner dependent on the charge of R57D. In summary, the ability for RNF168 to promote both this histone monoubiquitnation event and 53BP1 IRIF, but not self-accumulation into IRIF, correlates with proficient inhibition of HR.

### Expression of RNF168 WT and N221* can inhibit HR and promote 53BP1 IRIF in RNF8-deficient cells

Given that an N-terminal fragment of RNF168 can promote several DDR functions without itself accumulating into IRIF, we hypothesized that RNF168 function may not be entirely dependent on RNF8, which has been shown to promote RNF168 IRIF (5,6). RNF8 is a RING domain E3 ubiquitin ligase that localizes to DSBs via interaction with the DDR factor MDC1, and is important to promote IRIF of other downstream DDR factors, including BRCA1 and 53BP1 (5,6,14–17). However, 53BP1 IRIF have been shown to be partially restored in RNF8 depleted cells via RNF168-WT expression (6), which is consistent with our above hypothesis.

**Figure 4.**
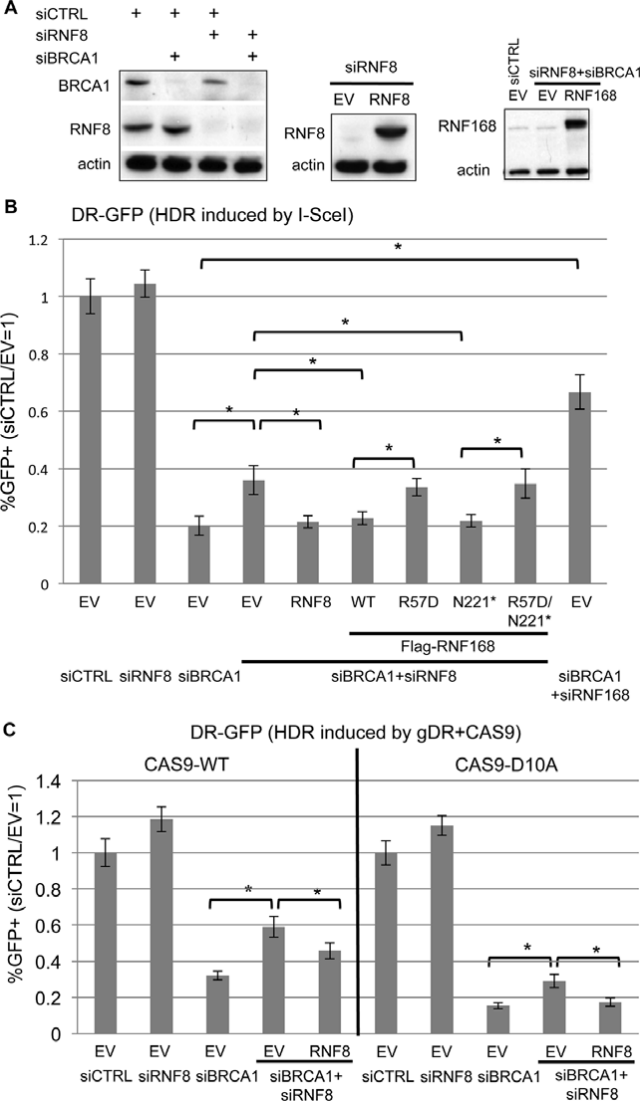
RNF8 depletion causes a modest increase in HR in BRCA1 deficient cells that can be reversed by expression of RNF168 WT and N221*. (**A**) Depletion of RNF8 and BRCA1 via siRNA and expression of RNF8. U2OS cells treated with siRNAs targeting BRCA1 and RNF8 (siBRCA1#7, siRNF8#5), and a non-targeting siRNA (siCTRL). Subsequent to siRNA treatment, cells were transfected with an expression vector for RNF8 (siRNF8#5-resistant), RNF168, or EV. Shown are immunoblot signals from transfections for RNF8, BRCA1, RNF168, and actin. (**B**) Analysis of HDR induced by I-SceI for cells depleted of BRCA1 and RNF8. The U2OS DR-GFP reporter cell line was treated with the siRNAs described in A, and subsequently co-transfected with the expression vector for I-SceI. In addition, using samples treated with siRNAs targeting BRCA1 and RNF8, each of the RNF168 expression vectors described in Figure 2, as well as RNF8, were included in the I-SceI transfection. Shown are the frequencies of *GFP+* cells for each reporter cell line, relative to parallel transfections with a non-targeting siRNA (siCTRL) and control EV. *P < 0.0001 (*n* = 6). (**C**) Analysis of HDR induced by CAS9 for cells depleted of BRCA1 and RNF8. Transfections were performed as in B, except replacing I-SceI with gRNA/CAS9 plasmids expressing gDR with CAS9-WT or CAS9-D10A, as shown in Figure 1. Frequencies of *GFP+* cells were measured as in B. *P ≤ 0.034 (*n* = 3).

To examine the influence of RNF168 on the DDR in RNF8-deficient cells, we first sought to characterize the effect of RNF8 depletion on HR in BRCA1 deficient cells. Previous studies have indicated that RNF8 may promote HR under certain circumstances. Namely, RNF8 is important for cellular resistance to replication stress, (41,42), meiosis (43), as well as HR in cells co-depleted of BRCA1 and 53BP1 (44). However, the influence of RNF8 depletion on HR in cells deficient in BRCA1 (i.e. without co-depletion of 53BP1) has been unclear. To test this, we depleted RNF8 and/or BRCA1 in the U2OS DR-GFP HDR reporter cell line (Figure 4A), and subsequently induced chromosomal breaks via I-SceI, CAS9-WT and CAS9-D10A (Figure 4B and C). From these experiments, we found that for each inducing nuclease, depletion of RNF8 alone did not obviously affect the frequency of HDR; whereas depletion of RNF8 in BRCA1 depleted cells caused a modest increase in HDR (≥1.8-fold, *P* < 0.005, Figure 4B and C), which was reversed for each nuclease via co-expression of siRNA-resistant RNF8 (Figure 4, *P* < 0.035). Thus, depletion of RNF8 can cause an increase in HDR in BRCA1 depleted cells, which is consistent in the role of RNF8 promoting 53BP1 IRIF (5,6,14–17). However, the modest effect of RNF8 depletion on HDR in this assay, particularly in comparison to the pronounced rescue of HDR caused by disruption of RNF168 or 53BP1 (Figure 4B) (8), is consistent with RNF8 also having a separate HR mediator role (41–44).

In any case, the finding that cells co-depleted of RNF8 and BRCA1 show elevated HDR, relative to BRCA1 depleted cells, provided an opportunity to address whether expression of RNF168 could inhibit HDR independently of RNF8. For this, we expressed different forms of RNF168 in cells co-depleted of RNF8 and BRCA1, and subsequently examined HDR. In these experiments, RNF168 is substantially overexpressed, based on immunoblotting analysis (>10-fold, Figure 4A). From these experiments, we found that RNF168-WT and RNF168-N221*, but not RNF168-R57D or RNF168-N221*/R57D, caused a significant reduction in HDR in cells depleted of RNF8 and BRCA1 (Figure 4B). In comparison to cells depleted of RNF8, while partial depletion of 53BP1 via siRNA causes an increase in HDR in BRCA1 depleted cells, co-transfection with the RNF168 expression vectors did not obviously inhibit HDR in these cells (Supplemental Figure S2A), which is consistent with the notion that RNF168 acts upstream of 53BP1 during inhibition of HR.

**Figure 5.**
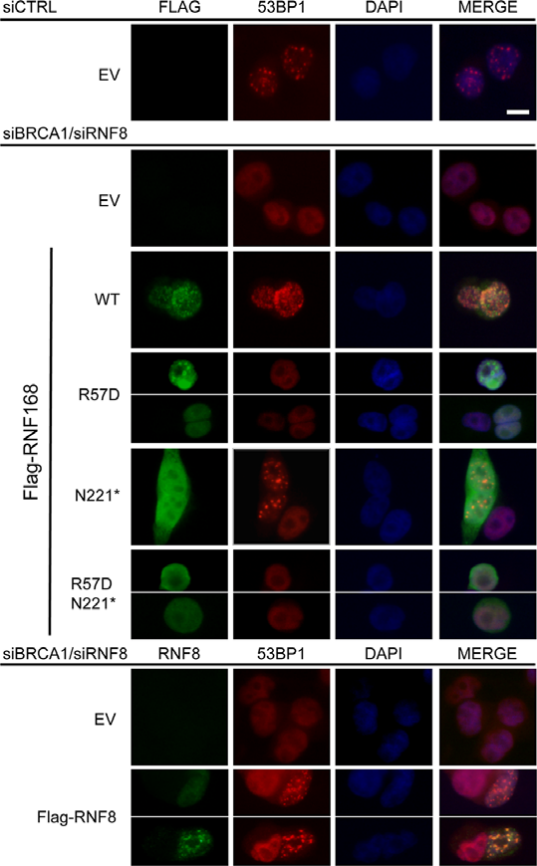
53BP1 IRIF can be restored in RNF8 depleted cells by expression of RNF168 WT and N221*. U2OS cells were treated with siCTRL or siBRCA1 and siRNF8 and subsequently transfected with a set of the Flag-tagged RNF168 expression vectors or the RNF8 expression vector, which are described in Figures 2A and 4A. Subsequently, cells were treated with 6 Gy of IR (Cs137), and allowed to recover for 4 h prior to fixation and immunostaining. RNF8 and 53BP1 antibodies were used for the RNF8 expression vector samples, and Flag and 53BP1 antibodies were used for the RNF168 expression vector samples. Shown are immunostaining, and DAPI staining images for representative cells from each transfection. Scale bar = 10 μm.

The above finding that RNF168 expression can inhibit HDR independently of RNF8 raised the possibility that RNF168-WT and RNF168-N221* expression might be proficient at promoting 53BP1 IRIF in cells depleted of RNF8. To test this, we co-depleted cells of RNF8 and BRCA1 to be consistent with the HDR reporter experiments, expressed RNF8 or the various forms of Flag-tagged RNF168, and examined IRIF of 53BP1. From these experiments, we found that cells co-depleted of RNF8 and BRCA1 failed to form 53BP1 IRIF, and that transient expression of RNF8 was able to restore 53BP1 IRIF (Figure 5), as expected (5,6,14–17). Furthermore, in cells co-depleted of RNF8 and BRCA1, we found that RNF168-WT itself formed IRIF and promoted 53BP1 IRIF; RNF168-N221* itself did not form IRIF but promoted 53BP1 IRIF (albeit at a 1.5-fold lower frequency than WT); whereas neither RNF168-R57D nor RNF168-N221*/R57D were proficient at promoting 53BP1 IRIF (Figure 5, Table 2). Notably, we found no obvious requirement for greater expression of N221* versus WT for promotion of 53BP1 IRIF, based on comparing WT and N221* cells with similar Flag immunostaining signals, as confirmed by quantifying the mean fluorescence intensity (Supplemental Figure S3). We also analyzed cells depleted of RNF8 alone (i.e. without BRCA1 depletion) and found similar results (Supplemental Figure S1). These experiments indicate that the functions of RNF168 during the DDR are not necessarily dependent upon RNF8, albeit under conditions of elevated expression of RNF168 (>10-fold, Figure 4A).

### Conserved motifs within RNF168-N221* are important to inhibit HDR

Finally, we sought to evaluate the effects of RNF168 R57D and N221* on HDR outside the context of BRCA1 depletion, and to examine the influence of conserved motifs within N221* on its HDR inhibition function. To begin with, we examined the influence of RNF168 on cells without BRCA1 depletion, as well as in cells depleted of another HR factor, FANCD2. FANCD2 is part of the Fanconi Anemia pathway that is important for DNA crosslink repair and HR (45,46). We have confirmed the role of FANCD2 in promoting HDR, in that depletion of FANCD2 via siRNA causes a decrease in the frequency of HDR, as measured by the DR-GFP assay (Supplemental Figure S2B). Next, we found that siRNF168 treatment alone, or combined with siFANCD2 treatment, caused an increase in HDR that was reversed by transient expression of RNF168 WT or N221*, but not R57D or R57D/N221* (Supplemental Figure S2B). Thus, in both BRCA1 proficient cells and FANCD2 depleted cells, RNF168-N221* appears proficient at inhibition of HDR, and the charge of R57 on RNF168 is important for this function.

**Figure 6.**
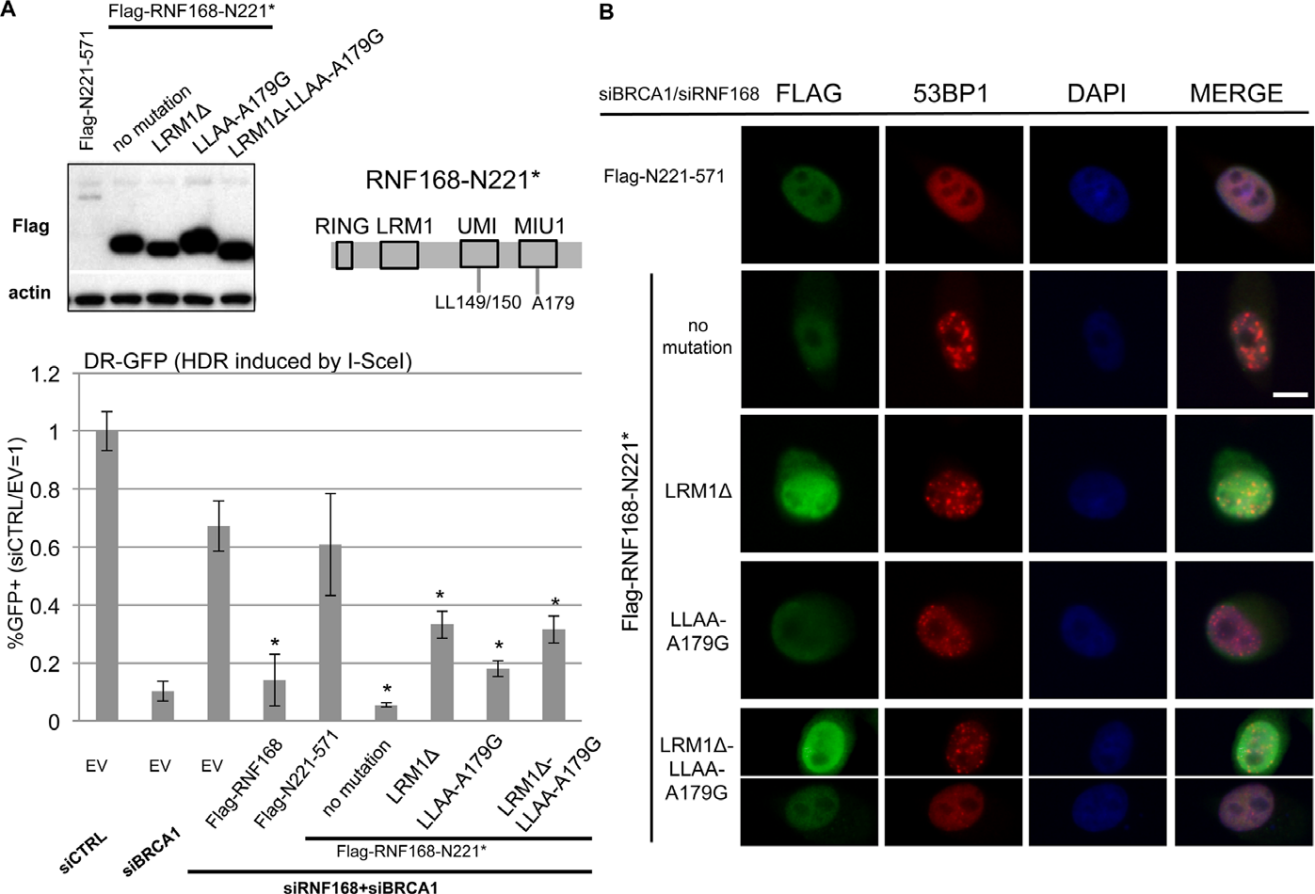
The LRM1 and ubiquitin binding motifs within RNF168-N221* are important but not essential for inhibition of HR. (**A**) Conserved motifs within N221* are important for inhibition of HDR. Shown is a diagram of N221* with the approximate positions of the LRM1 and UMI/MIU1 motifs. Also shown are immunoblotting signals for Flag and actin for cells transfected with a set of expression plasmids for Flag-RNF168-N221* with mutations in these conserved motifs, as well as Flag-RNF168-N221-571. U2OS DR-GFP cells were treated with siRNAs targeting BRCA1 and RNF168, and subsequently co-transfected with expression vectors for I-SceI and a set of RNF168 mutants. Shown are the frequencies of *GFP+* cells for each reporter cell line, relative to parallel transfections with a non-targeting siRNA (siCTRL) and control EV. *distinct from EV, *P* < 0.0001 (*n* = 6). (**B**) RNF168 mutants that show at least intermediate inhibition of HR are also proficient for promoting 53BP1 IRIF. U2OS cells were treated with siCTRL or siBRCA1 and siRNF168 and subsequently transfected with Flag-tagged RNF168 expression vectors described in A. Subsequently, cells were treated with 6 Gy of IR (Cs137), and allowed to recover for 4 h prior to fixation and immunostaining. Shown are Flag and 53BP1 immunostaining, and DAPI staining images for representative cells from each transfection. The exposure times for each type of immunostain are the same for each cell. The cells were selected to represent the 53BP1 IRIF results for each RNF168 mutant as quantified in Table 2, but do not necessarily represent the average intensity of Flag staining for each RNF168 mutant, which can show variability among cells. Scale bar = 10 μm.

In addition to the R57 residue in the RING domain, the N221* fragment of RNF168 contains other conserved motifs, which we sought to evaluate for their influence on the inhibition of HDR. Two motifs that are present within N221* have been shown to be functionally redundant in promoting ubiquitin binding of RNF168: UMI (UIM- and MIU-related ubiquitin binding domain) and MIU1. Previous studies have found that mutation of two conserved leucines in the UMI domain (L149A L150A; LLAA) combined with mutation of a conserved alanine residue in MIU1 (A179G) along with a similar mutation in MIU2 at the RNF168 C-terminus, causes a loss of ubiquitin binding and loss of RNF168 IRIF (25). Thus, to test the role of the ubiquitin binding motifs in the inhibition of HDR, we generated the N221* LLAA/A179G mutant. An additional conserved motif has been identified between the RING domain and UMI/MIU1: the LR motif (LRM1), which contains a leucine arginine (L116 R117) dipeptide (18). LRM1 does not appear to be required for ubiqtuitin binding of RNF168, but rather appears to be important for accumulation of RNF168 into IRIF (18). Thus, we also generated an expression vector for N221* with an LRM1 deletion mutation (ΔLRM1; ΔG114-V127 mutation), as well as a combination of LRM1 deletion with the UMI/MIU1 mutations (ΔLRM1/LLAA/A179G). Finally, we generated an expression vector for the C-terminus of RNF168 (N221-571) to formally test the prediction that this fragment is deficient at inhibition of HDR. As in previous experiments, each mutant contains a Flag immunotag at the N-terminus, and we confirmed their expression using a Flag immunoblot, although N221-571 was poorly expressed (Figure 6A).

We then examined each of these mutants in the DR-GFP assay for HDR in cells co-depleted of BRCA1 and RNF168. From these experiments, each of the N221* mutants showed an intermediate inhibition of HDR (Figure 6A). Namely, the ΔLRM1 and ΔLRM1/LLAA/A179G mutants of N221* inhibited HDR 2-fold, which is distinct from both WT (5-fold, *P* < 0.002) and N221* (12-fold, *P* < 0.0001). The LLAA/A179G mutant of N221* inhibited HDR 3.7-fold, which is significantly less than N221* (*P* < 0.0001), but not WT. Consistent with the notion that these mutant forms show at least intermediate inhibition of HR, we found that each were able to promote 53BP1 IRIF (Figure 6B, Table 2). Although, the frequency of 53BP1 IRIF positive cells was reduced 40% for the ΔLRM1/LLAA/A179G mutant, relative to both WT and N221* (*P* = 0.0003 and *P* < 0.0001, respectively, Table 2).

In contrast to each of these N221* mutants, the N221-571 C-terminal RNF168 expression vector caused no statistical difference in the frequency of HDR relative to the control EV (Figure 6A). Of course, this experiment does not eliminate the possibility that if N221-571 could be expressed at higher levels, it might show activity to inhibit HR. However, even though N221-571 showed overall low expression by immunoblotting analysis (Figure 6A), it was nevertheless possible to identify cells with detectable levels of Flag staining of N221-571 for 53BP1 IRIF analysis (Figure 6B). From this analysis, we found that cells expressing N221-571 were unable to promote 53BP1 IRIF (Figure 6B, Table 2), similar to above findings with the R57D mutants. These findings indicate that the LRM1 and ubiquitin binding motifs within an N-terminal fragment of RNF168 are important, but not essential, for inhibition of HDR. In summary, we suggest that accumulation of RNF168 into IRIF is not essential for several RNF168 functions during the DDR, whereas the charge of residue R57 is critical for these functions.

## DISCUSSION

We have investigated the function of RNF168 during the inhibition of HR repair of chromosomal breaks in BRCA1 deficient cells, finding that the charge of residue R57, but not the C-terminal 60% of RNF168 (residues 221-571), is required for this function. We also present evidence that the N-terminal 220 residues of RNF168 (1-220/N221*) are proficient at promoting 53BP1 IRIF and K13/15 ubiquitination of H2AX, in a manner dependent on the charge of R57. Using purified proteins, the RNF168-R57D mutant has been shown to be proficient for ubiquitin ligase activity, but is deficient at the monoubiquitination of nucleosomal H2A/H2AX at K13/15 (11). Thus, our findings indicate that this histone ubiquitination event is important for inhibition of HR in BRCA1 deficient cells, but of course does not exclude a role for other ubiquitination events that may require RNF168-R57. In addition, we found that an N-terminal fragment of RNF168 (N221*), which is proficient for inhibition of HR, was deficient at accumulation into IRIF. As well, the LRM1 and ubiquitin binding motifs within this N-terminal fragment of RNF168 are important, but not essential, for inhibition of HR. Together, these findings indicate that focal accumulation of RNF168 is not essential for inhibition of HR, whereas the ability of RNF168 to promote H2AX monoubiquitination and 53BP1 recruitment to DSBs correlates with its HR inhibition function.

Regarding a possible temporal order of events of RNF168-mediated inhibition of HR, our data are consistent with a model whereby RNF168 promotes H2A/H2AX monoubiquitination on K13/15 on nucleosomes near DSBs, which creates a binding site on these nucleosomes for the HR inhibitory factor 53BP1 (9,10,13). In support of this model, recent studies have identified an acidic patch on H2A/H2B dimers important for RNF168-mediated ubiquitination of nucleosomes (47,48). We suggest that such RNF168-mediated signaling events that are important to inhibit HR do not require focal accumulation of RNF168 at DSBs.

The above model underscores the notion that focal accumulation of repair factors may not necessarily be required for DSB repair, but does not exclude the possibility that such focal accumulation may have other functions during the DDR. Focus formation likely reflects not only recruitment of a factor to DSBs, but also accumulation of a significant amount of the factor at a high-localized concentration that may include substantial spreading along chromatin, as well as retention of such recruitment over time. Indeed, focal retention may persist long after repair is completed. For example, both formation and clearance of γH2AX IRIF show slower kinetics compared to IR-induced DSB induction and repair as measured by chromosome integrity using pulse field gel electrophoresis (49). As well, DSBs are not necessarily required for focal accumulation of DDR factors, in that γH2AX foci can be induced by targeting other DDR factors (e.g. NBS1) to chromatin (50). However, apart from DSB repair *per se*, restoration of the undamaged chromatin state is also a likely important aspect of the DDR (51), including the removal of γH2AX by phosphatases and/or nucleosome remodeling factors (20). As another example, DSBs can induce transcriptional silencing at neighboring regions (52), which likely requires reversal after repair. Accordingly, while focal accumulation of RNF168, which likely reflects substantial and persistent localization at damaged chromatin, does not appear essential for inhibition of HR during DSB repair, such focal accumulation of RNF168 may nevertheless be important for other aspects of the DDR.

The finding that RNF168 focal accumulation, which is promoted by RNF8 (5,6), is not essential for its function to inhibit HR supports the notion that RNF168 and RNF8 have overlapping but distinct roles during HR. The role of RNF8 is similar to RNF168 in that both factors are important for 53BP1 IRIF, and all three of these factors promote class switch recombination during antibody maturation (2,7,43,53). As well, we present evidence that RNF8 depletion can partially suppress the HR defects caused by BRCA1 depletion. However, the degree of HR suppression caused by RNF8 depletion is significantly less than for RNF168 depletion, which supports a distinct and complex role for RNF8 during HR. Consistent with a distinct HR mediator function, RNF8 has been shown to promote cellular resistance to replication stress, and is important for meiosis (41–44,54). Furthermore, RNF8 is not absolutely required for RNF168-mediated DDR signaling, in that expression of RNF168 has been shown to rescue 53BP1 IRIF in RNF8 depleted cells (6), and using purified proteins, RNF168 was shown to be proficient at ubiquitinating nucleosomal H2A/H2AX without RNF8 (11). Additionally, we have found that expression of RNF168 WT or N221*, but not the R57D forms of RNF168, can rescue the effects of depletion of RNF8 on HR and 53BP1 IRIF. In summary, we suggest that while the role of RNF8 for promoting IRIF of RNF168 and 53BP1 may partially contribute to inhibition of HR in BRCA1 deficient cells, RNF8 also likely plays an independent HR mediator role, and RNF168 function during HR inhibition is not absolutely dependent on RNF8.

As part of our study, we have applied the gRNA/CAS9 system to examine repair of distinct inducing nucleases. We examined both CAS9-WT that induces blunt ended DSBs, and CAS9-D10A that is deficient at second strand cleavage (33,34,37). From a comparison with I-SceI, we find that CAS9-WT is more efficient at inducing mutagenic EJ than HDR. The reason for this distinction is unclear, but perhaps the DNA strand displaced by the gRNA via CAS9 is prone to end degradation prior to EJ. Even though CAS9-WT is proficient at promoting mutagenic EJ, we find that expression of the non-processive exonuclease Trex2 can further increase the frequency of such events. Trex2 expression has been shown to promote mutagenic EJ during DSB repair induced by other nucleases, including I-SceI and TALENs (39,55), and accordingly can enhance the frequency of deletion mutations during DSB-induced genome engineering. Our findings indicate that this Trex2 approach can be applied to DSBs induced by gRNA/CAS9-WT, and thereby enhance mutation induction via this technology. In contrast to CAS9-WT, we find that CAS9-D10A is relatively more efficient at inducing HDR versus EJ, which is consistent with the known deficiency of this enzyme for second-strand cleavage, such that this mutant has been referred to as a nickase (33,34,37). Though, the ability for CAS9-D10A to induce EJ events, albeit at a much lower frequency than CAS9-WT, indicates that either the nicks induced by CAS9-D10A are processed into DSBs, and/or that CAS9-D10A retains some low level of second strand cleavage activity, as has been proposed in other reports (33,37). In any case, using these distinct nucleases, we present evidence that the influence of BRCA1 and RNF168 during HDR is not limited to one type of DSB inducing lesion, and hence may reflect a universal feature of mammalian HDR. In summary, we suggest that RNF168-mediated DDR signaling that is dependent on R57, but not its own focal accumulation at DNA damage, is important for inhibition of HR in BRCA1 deficient cells.

## SUPPLEMENTARY DATA

Supplementary Data are available at NAR Online.

SUPPLEMENTARY DATA
