# Supplementary material for: An RNF168 fragment defective for focal accumulation at DNA damage is proficient for inhibition of homologous recombination in BRCA1 deficient cells
Source: Nucleic Acids Res. 2014 May 14;42(12):7720–33. doi: 10.1093/nar/gku421 (PMC4081061; doi:10.1093/nar/gku421)
Supplement: SUPPLEMENTARY DATA [file supp_42_12_7720__index.html]

An RNF168 fragment defective for focal accumulation at DNA damage is proficient for inhibition of homologous recombination in BRCA1 deficient cells — SUPPLEMENTARY DATA 

# An RNF168 fragment defective for focal accumulation at DNA damage is proficient for inhibition of homologous recombination in BRCA1 deficient cells

## SUPPLEMENTARY DATA

**Files in this Data Supplement:**

- SUPPLEMENTARY DATA
