# Supplementary material for: An RNF168 fragment defective for focal accumulation at DNA damage is proficient for inhibition of homologous recombination in BRCA1 deficient cells
Source: Nucleic Acids Res. 2014 May 14;42(12):7720–33. doi: 10.1093/nar/gku421 (PMC4081061; doi:10.1093/nar/gku421)

SUPLEMENTAL DATA FIGURE LEGENDS

Supplemental Figure S1. RNF168 WT and N221* can promote 53BP1 IRIF in cells depleted of RNF168 or RNF8 alone, without BRCA1 co-depletion. U2OS cells were treated with siRNAs targeting RNF168 or RNF8, and subsequently transfected with RNF168 expression vectors. Cells were treated with IR and examined by immunofluorescence, as described in Figs 3 and 5. Shown are immunostaining and DAPI staining images for representative cells from each transfection. Scale bar = 10 μm.

Supplemental Figure S2. Effects of RNF168 WT, N221*, and R57D mutants on HDR in other contexts. A. Expression of RNF168 WT and N221* does not show obvious effects on HDR in cells depleted of 53BP1 and BRCA1. The U2OS DR-GFP reporter cell line was treated with the siRNAs targeting 53BP1 and/or BRCA1, and subsequently co-transfected with the expression vector for I-SceI. Also included in the I-SceI transfection were expression vectors for RNF168 described in Fig 2. *$p<0.0001$ ($n=6$). Also shown is immunoblotting analysis of 53BP1 and actin of U2OS cells treated with si53BP1 or siCTRL. **B.** Expression of RNF168 WT and N221* inhibits HDR in BRCA1 proficient cells and FANCD2 deficient cells. The U2OS DR-GFP reporter cell line was treated with the siRNAs targeting FANCD2 and/or RNF168. These cells were subsequently co-transfected with I-SceI and RNF168 expression vectors, as described in A. Shown are the frequencies of *GFP+* cells for each reporter cell line, relative to parallel transfections with a non-targeting siRNA (siCTRL) and control EV. *$p<0.0001$ ($n=6$). Also shown is immunoblotting analysis of FANCD2, RNF168, and actin of U2OS cells following siRNA treatment.

Supplemental Figure S3. Similar expression levels of RNF168-N221* versus WT promote 53BP1 IRIF, based on analysis of Flag staining intensity. As described in Fig 5, cells were co-treated with siBRCA1 and siRNF8, transfected with Flag-RNF168, were treated with IR, and examined by immunofluorescence. Shown are representative individual cells that express RNF168-N221* and RNF168-WT at similar levels, based on quantification of the mean fluorescence intensity of the Flag immunostain, which is shown to the left (MFI, relative luminosity units quantified using ImagePro Premier). Scale bar = 10 μm.

Supplemental Figure S1

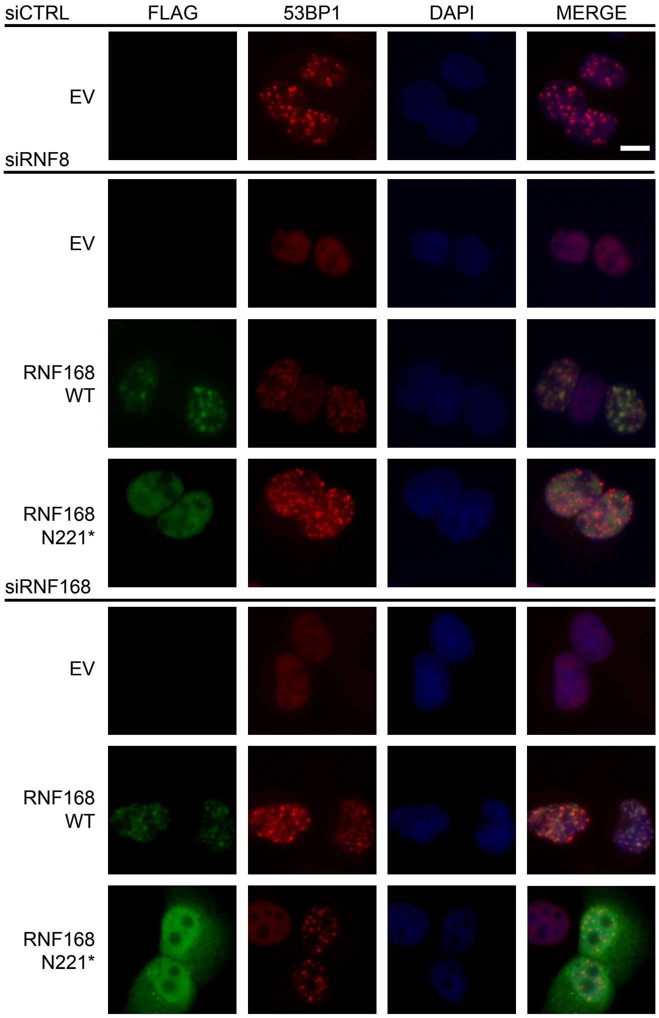

Supplemental Figure S2

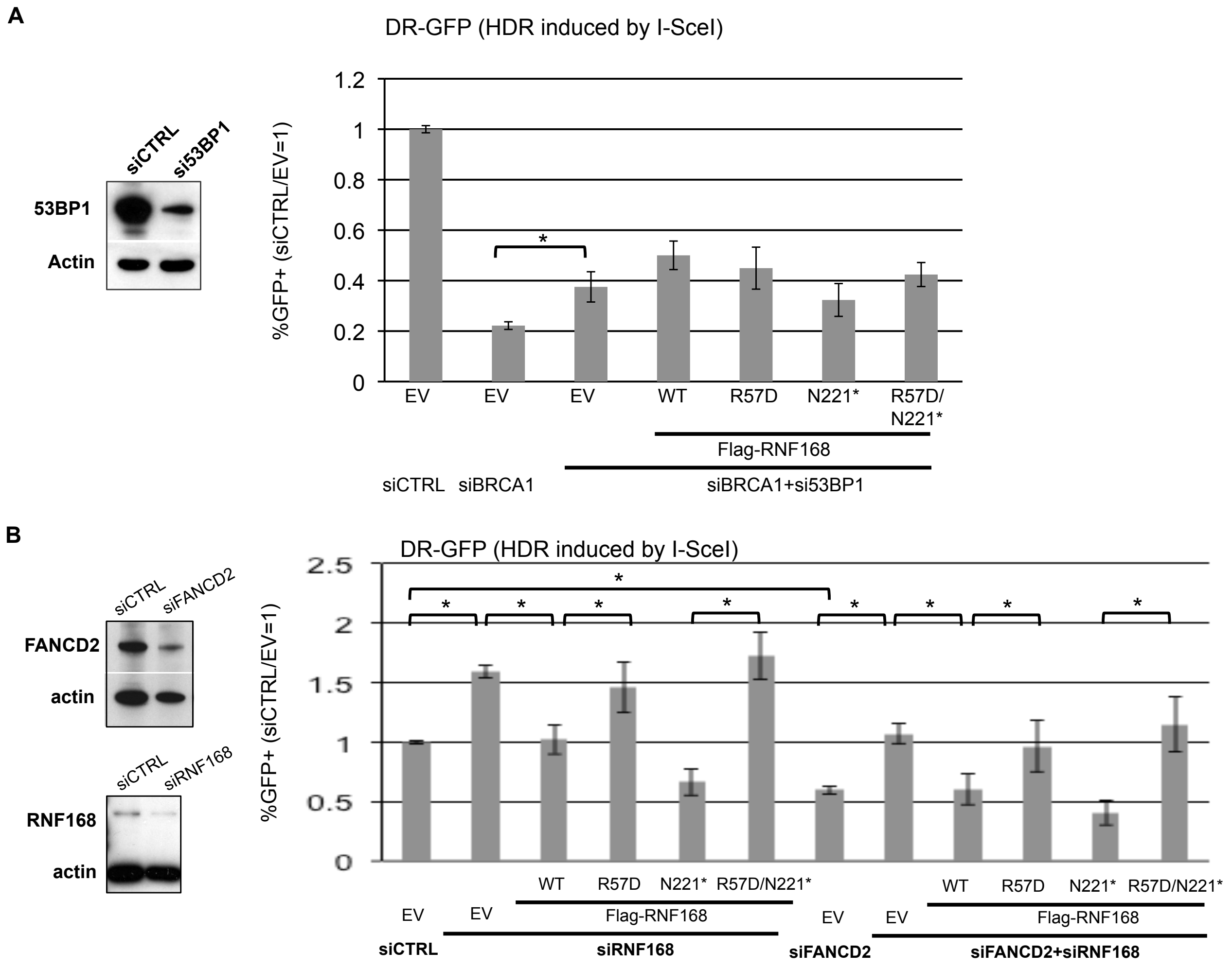

Supplemental Figure S3

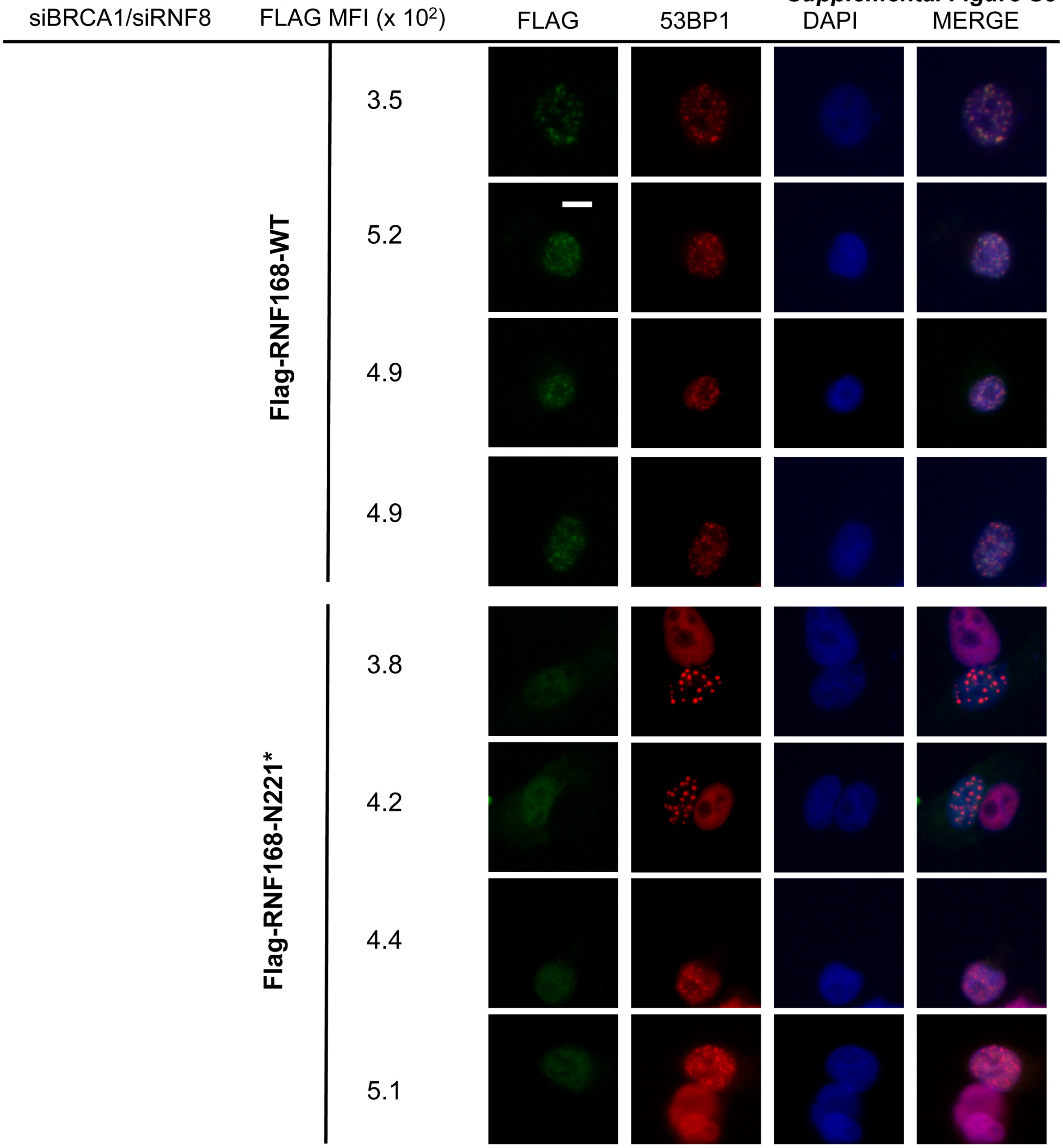

Supplement: SUPPLEMENTARY DATA [file supp_gku421_nar-00135-d-2014-File008.pdf]
